# Skin Comfort Sensation with Mechanical Stimulus from Electronic Skin

**DOI:** 10.3390/ma17122920

**Published:** 2024-06-14

**Authors:** Dongcan Ji, Yunfan Zhu, Min Li, Xuanqing Fan, Taihua Zhang, Yuhang Li

**Affiliations:** 1 Institute of Solid Mechanics, Beihang University (BUAA), Beijing 100191, China; 2 International Innovation Institute, Beihang University (BUAA), Yuhang District, Hangzhou 311115, China; 3 Aerospace Information Research Institute, Chinese Academy of Sciences, Beijing 100094, China; 4 Aircraft and Propulsion Laboratory, Ningbo Institute of Technology, Beihang University (BUAA), Ningbo 315100, China

**Keywords:** electronic skin, skin pain sensation, mechanical stimulus, mechanical model of skin

## Abstract

The field of electronic skin has received considerable attention due to its extensive potential applications in areas including tactile sensing and health monitoring. With the development of electronic skin devices, electronic skin can be attached to the surface of human skin for long-term health monitoring, which makes comfort an essential factor that cannot be ignored in the design of electronic skin. Therefore, this paper proposes an assessment method for evaluating the comfort of electronic skin based on neurodynamic analysis. The holistic analysis framework encompasses the mechanical model of the skin, the modified Hodgkin–Huxley model for the transduction of stimuli, and the gate control theory for the modulation and perception of pain sensation. The complete process, from mechanical stimulus to the generation of pain perception, is demonstrated. Furthermore, the influence of different factors on pain perception is investigated. Sensation and comfort diagrams are provided to assess the mechanical comfort of electronic skin. The comfort assessment method proposed in this paper provides a theoretical basis when assessing the comfort of electronic skin.

## 1. Introduction

The term ‘electronic skin’ is used to describe electronic devices that possess the properties of human skin, in addition to other functions [1,2,3]. These flexible electronic devices are characterized by high stretchability and high toughness [4,5,6], enabling them to conform to the contours of the human body and withstand complex deformations and vibrations [7,8,9,10,11]. In addition to mimicking the tactile sensing functions of human skin [12,13,14,15,16,17], electronic skin can also detect other physical and chemical information, including analyzing lactate [18,19], glucose [20,21], ethanol [22,23], and electrolytes [19,24,25] in biofluids such as sweat and tears [26], and monitoring electrophysiological signals including electrocardiography (ECG) [27,28,29,30], electromyography (EMG) [31], and electroencephalography (EEG) [32], enabling health monitoring and disease diagnosis.

The development of electronic skin has led to the necessity of long-term conformally attached devices to collect physiological information [31,33,34], which has highlighted the importance of comfort in the design of electronic skin [35]. The uncomfortable sensations induced by electronic skin can be attributed to the mechanical [36,37,38,39], thermal [40,41,42,43,44,45,46], and electrical [47,48] stimuli during operation. On the one hand, these stimuli comprise the on-demand therapeutics applied by the electronic skin, which is able to reduce long-term medical costs and health risks. On the other hand, the stimuli come from the inevitable accumulation of Joule heat and mechanical differences compared to human skin [49].

A significant number of researchers have concentrated their efforts on the comfort of electronic skin, conducting investigations from a variety of vantage points. Some researchers attempted to bridge the gap between electronic skin devices and human skin by modifying the structural design [36,37,50,51,52]. The utilization of the horseshoe topology and the control of the geometric parameters of the structure has enabled the minimization of the mechanical difference between electronic skin and human skin, resulting in the realization of mechanical stealth during the working process of the electronic skin, and has effectively improved the comfort perceived by the human skin. Other researchers have studied the sensations experienced by the skin under thermo-mechanical coupling stimuli from electronic skin, proposing a thermal comfort assessment method consisting of a stimulation and perception evaluation [53,54,55,56,57,58,59,60,61]. This method allows for the quantification of the sensation of pain perceived by the skin under the combined action of mechanical, thermal, and chemical stimuli caused by the electronic skin. However, there is a paucity of studies analyzing the comfort of electronic skin considering mechanical stimuli.

This paper presents an assessment method for evaluating comfort when wearing electronic skin devices. The method encompasses the mechanical model of skin, the transduction model of stimuli, and the modulation and perception model of pain sensation. The mechanical model of skin is established to obtain the theoretical stress distribution, which is verified through comparison using the finite element analysis (FEA). Subsequently, the nerve impulse under mechanical stimuli is acquired through the modified Hodgkin–Huxley model, and the sensation is evaluated based on a mathematical model of gate control theory. Additionally, the factors that influence the comfort of the electronic skin, including the magnitude of mechanical stimuli and the location of the nociceptor, are also discussed.

## 2. Analysis of Skin Sensation

**Figure 1:**
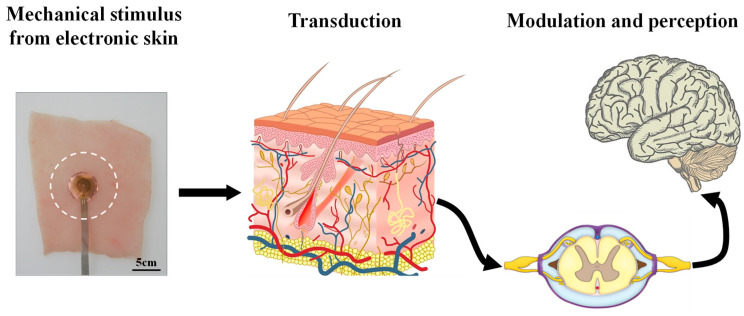
Schematic of the skin sensation procedure, which is induced by the mechanical stimulus caused by electronic skin: a thermal protecting substrate [41] is transduced to the nervous impulses by the nociceptor on the skin, and then modulated and perceived by the central nervous system.

The process of skin sensations is schematically illustrated in Figure 1. A sensation is induced by the mechanical stimuli from the thermal management electronic skin device, namely, a thermal protecting substrate [41], and transduced to the nervous impulses by the nociceptors on the skin. The signal is then transmitted to the central nervous system, where it is modulated and precepted by the mid-brain and spinal cord. The following section will present a detailed analysis of each step in this process.

### 2.1. Stress Distribution on the Skin

**Figure 2:**
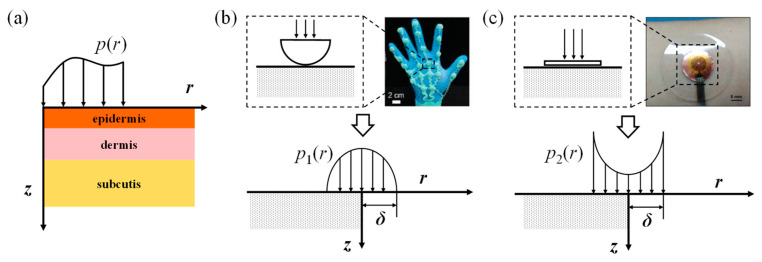
(**a**) The mechanical model of skin, which consists of three layers: the epidermis, dermis and subcutis. Two types of mechanical loading are analyzed, including (**b**) a hemispherical mechanical stimulus *p*_1_(*r*) induced by the wearable bilateral-vibrotactile-actuator-based virtual reality interface targeting the palms [63], and (**c**) the mechanical stimulus induced by the thermal protecting substrate *p*_2_(*r*) [41].

As the largest organ of the human body, skin generally consists of three layers: the epidermis, dermis, and subcutis. For the simplicity of analysis, each layer is considered to be isotropic [62]. Considering the mechanical loading applied by the electronic skin, an axisymmetric model of skin is established, as illustrated in Figure 2a. The mechanical loading from electronic skin is denoted by *p*(*r*). The zero-displacement boundary condition is assumed at the bottom surface of the skin. The stress components satisfy the equilibrium equations and constitutive equations:

(1) $$\left\{\begin{array}{c} \frac{\partial \sigma_{r}}{\partial r}+\frac{\partial \tau_{zr}}{\partial z}+\frac{\sigma_{r}-\sigma_{\theta }}{r}=0\\ \frac{\partial \sigma_{z}}{\partial z}+\frac{\partial \tau_{zr}}{\partial r}+\frac{\tau_{zr}}{r}=0, \end{array}\right.$$

(2) $$\left\{\begin{array}{c} \sigma_{r}=\left(\lambda +2G\right)\frac{\partial u}{\partial r}+\frac{\lambda }{r}u+\lambda \frac{\partial w}{\partial z}\\ \sigma_{\theta }=\lambda \frac{\partial u}{\partial r}+\left(\lambda +2G\right)\frac{u}{r}+\lambda \frac{\partial w}{\partial z}\\ \sigma_{z}=\lambda \frac{\partial u}{\partial r}+\lambda \frac{u}{r}+\left(\lambda +2G\right)\frac{\partial w}{\partial z}\\ \tau_{zr}=G\left(\frac{\partial u}{\partial z}+\frac{\partial w}{\partial r}\right), \end{array}\right.$$

Here, *λ* and *G* are the Lame constants, which are linked with the Young’s modulus *E* and Poisson’s ratio *ν* by the following:

(3) $$\lambda =\frac{\nu E}{\left(1+\nu \right)\left(1-2\nu \right)},\;G=\frac{E}{2\left(1+\nu \right)}.$$

Setting u* = 2*Gu*, w* = 2*Gw* and utilizing the Hankel transform leads to the following:

(4) $$\frac{d\hat{\Lambda }}{dz}=\frac{d}{dz}\left[\begin{array}{c} \hat{u}*\\ \hat{w}*\\ {\hat{\sigma }}_{z}\\ {\hat{\tau }}_{zr} \end{array}\right]=\left[\begin{array}{cccc} 0 & \xi & 0 & 2\\ -\frac{\nu \xi }{1-\nu } & 0 & \frac{1-2\nu }{1-\nu } & 0\\ 0 & 0 & 0 & -\xi \\ \frac{\xi^{2}}{1-\nu^{2}} & 0 & \frac{\nu \xi }{1-\nu } & 0 \end{array}\right]\left[\begin{array}{c} \hat{u}*\\ \hat{w}*\\ {\hat{\sigma }}_{z}\\ {\hat{\tau }}_{zr} \end{array}\right]=\Phi \left(\xi \right)\hat{\Lambda },$$

(5) $$\left\{\begin{array}{c} \hat{u}*\left(\xi ,z\right)=H_{1}\left[u*\left(r,z\right)\right]=\int_{0}^{\infty }rf\left(r,\;z\right)J_{1}\left(\xi r\right)dr=\hat{f}\left(\xi ,\;z\right),\\ \hat{w}*\left(\xi ,z\right)=H_{0}\left[w*\left(r,z\right)\right]=\int_{0}^{\infty }rf\left(r,\;z\right)J_{0}\left(\xi r\right)dr=\hat{f}\left(\xi ,\;z\right),\\ {\hat{\sigma }}_{z}\left(\xi ,z\right)=H_{0}\left[\sigma_{z}\left(r,z\right)\right]\\ {\hat{\tau }}_{zr}\left(\xi ,z\right)=H_{1}\left[\tau_{zr}\left(r,z\right)\right]. \end{array}\right.$$

In this paper, *H_i_* and *H_i_*^−1^ are the i-order Hankel transform and reversed Hankel transform. The solution of Equation (4) is as follows:

(6) $$\hat{\Lambda }\left(\xi ,z\right)=\exp\left[\Phi \left(\xi \right)z\right]\hat{\Lambda }\left(\xi ,0\right)=\Psi \left(\xi ,z\right)\hat{\Lambda }\left(\xi ,0\right),$$

The matrix Ψ(*ξ*, *z*) is detailed in Appendix A. Considering that the Lame constants are different in each layer of skin, u* and w* are substituted with *u* and *w* in Equation (6):

(7) $$\left[\begin{array}{c} \hat{u}\left(\xi ,z\right)\\ \hat{w}\left(\xi ,z\right)\\ {\hat{\sigma }}_{z}\left(\xi ,z\right)\\ {\hat{\tau }}_{zr}\left(\xi ,z\right) \end{array}\right]=\overset{\text{\textasciicircum{}}}{Y}\left(\xi ,z\right)=N\left(\xi ,\;z\right)\overset{\text{\textasciicircum{}}}{Y}\left(\xi ,0\right),$$

The matrix *N*(*ξ*, *z*) is detailed in Appendix B. Due to the fact that each layer of skin has different mechanical properties, Equation (7) becomes:

(8) $$\overset{\text{\textasciicircum{}}}{Y}\left(\xi ,z\right)=\prod_{i=1}^{3}N\left(E_{i},\;\nu_{i},\;\xi ,\;z\right)\overset{\text{\textasciicircum{}}}{Y}\left(\xi ,0\right)=\bar{N}\left(\xi ,\;z\right)\overset{\text{\textasciicircum{}}}{Y}\left(\xi ,0\right).$$

*σ_z_* and *τ_zr_* can be obtained by utilizing the reversed Hankel transform. The other stress components, except *σ_z_* and *τ_zr_*, can be obtained through the constitutive equations. The complete $\hat{Y}\left(\xi ,0\right)$ is obtained by utilizing the zero-displacement boundary condition at the bottom surface of the skin:

(9) $$\left[\begin{array}{c} \hat{u}\left(\xi ,0\right)\\ \hat{w}\left(\xi ,0\right) \end{array}\right]=-{\left[\begin{array}{cc} {\bar{N}}_{11}\left(\xi ,\;H\right) & {\bar{N}}_{12}\left(\xi ,\;H\right)\\ {\bar{N}}_{21}\left(\xi ,\;H\right) & {\bar{N}}_{22}\left(\xi ,\;H\right) \end{array}\right]}^{-1}\left[\begin{array}{cc} {\bar{N}}_{13}\left(\xi ,\;H\right) & {\bar{N}}_{14}\left(\xi ,\;H\right)\\ {\bar{N}}_{23}\left(\xi ,\;H\right) & {\bar{N}}_{24}\left(\xi ,\;H\right) \end{array}\right]\left[\begin{array}{c} \hat{p}\left(\xi \right)\\ 0 \end{array}\right],$$

where $\hat{p}\left(\xi \right)$ is the 0-order Hankel transform of *p*(*r*).

As mentioned before, the *p*(*r*) is the mechanical stimulus induced by the electronic skin. The electronic skins have different forms according to their different functions, leading to different mechanical loadings on the skin. Two typical electronic skin devices were taken as examples, as demonstrated in Figure 2b,c. Figure 2b shows a wearable bilateral vibrotactile (BV) actuator-based virtual reality (VR) interface targeting the palms [63]. The hemispherical BV actuator is tightly mounted on the skin, and the mechanical stimulus is denoted by *p*_1_(*r*) in this paper. *p*_1_(*r*) and ${\hat{p}}_{1}\left(\xi \right)$ can be expressed as follows:

(10) $$\begin{array}{l} p_{1}(r)=\left\{\begin{array}{cc} \frac{3p}{2}\sqrt{1-\frac{r^{2}}{\delta^{2}}} & \left(r\leq \delta \right)\\ 0 & \left(r>\delta \right) \end{array}\right.,\\ {\hat{p}}_{1}\left(\xi \right)=\frac{3p\delta }{2\xi }\frac{\sin\xi \delta -\xi \delta \cos\xi \delta }{{\left(\xi \delta \right)}^{2}}. \end{array}$$

Figure 2c demonstrates a thermal protecting substrate, consisting of a phase change material with a metal film on the top of the soft polymer [41]. The mechanical stimulus from the planar-shaped thermal protecting substrate is denoted as *p*_2_(*r*). According to our previous research [64], the mechanical stimulus induced by the rigid metal film *p*_2_(*r*) and corresponding Hankel transform ${\hat{p}}_{2}\left(\xi \right)$ can be expressed as follows:

(11) $$\begin{array}{l} p_{2}(r)=\left\{\begin{array}{cc} \frac{p}{2}\frac{1}{\sqrt{1-\frac{r^{2}}{\delta^{2}}}} & \left(r\leq \delta \right)\\ 0 & \left(r>\delta \right) \end{array}\right.,\\ {\hat{p}}_{2}\left(\xi \right)=\frac{p\delta \sin\xi \delta }{2\xi }. \end{array}$$

### 2.2. Model of Transduction

Nervous impulses originate from the current, which is induced by the opening of the ion channels of the nociceptors. According to previous research [58], the ion channels are generally gated by three kinds of stimuli: thermal, mechanical, and chemical stimuli. The total stimulus-induced current consists of three components:

(12) $$I_{\mathrm{st}}=I_{\mathrm{heat}}+I_{\mathrm{mech}}+I_{\mathrm{chem}}.$$

All of these current components need to be considered in the analysis of thermo-mechanical coupling stimuli to account for the effect of temperature, thermal stress, and thermal burn damage, respectively. For the skin sensations encountered under mechanical stimuli, the temperature change can be ignored [65]; thus, only the *I*_mech_ needs to be considered:

(13) $$I_{\mathrm{mech}}=\left[C_{m1}\exp(\frac{\sigma_{\max}-\sigma_{t}}{C_{m2}\sigma_{t}})+C_{m3}\right]\times H(\sigma_{\max}-\sigma_{t}),$$

Here, *H*(*x*) is the Heaviside function. Three mechanical-stimulus-related parameters are given by *C*_m1_ = 1 μA/cm^2^, *C*_m2_ = 3, and *C*_m3_ = −1 μA/cm^2^. The mechanical threshold of pain sensation is given by *σ*_t_ = 0.2 MPa [54]. Here, *σ*_max_ represents the stress level on the nociceptor, which is defined as follows:

(14) $$\sigma_{\max}=\max\{\left|\sigma_{r}\right|,\left|\sigma_{z}\right|\}.$$

**Figure 3:**
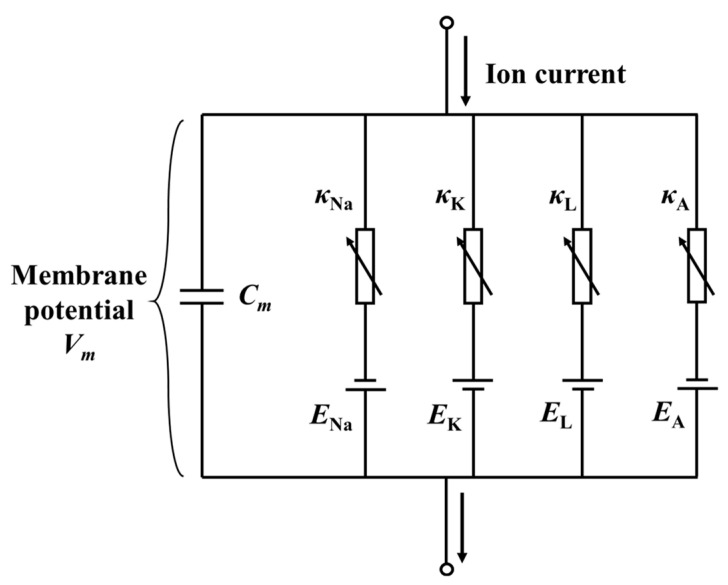
The schematic of the modified Hodgkin and Huxley model. Different ionic current components, including *I*_Na_, *I*_K_, *I*_L_, and *I*_A_, are related to the membrane potential *V*_m_ and corresponding reversal ionic potentials and ionic conductances [54].

The intensity of the external stimulus is determined through the frequency of the nerve impulses. The modified Hodgkin–Huxley (H–H) model is utilized to describe the generation of action potentials [54], which is schematically shown in Figure 3:

(15) $$C_{m}\frac{dV_{m}}{dt}=I_{\mathrm{mech}}+I_{\mathrm{shift}}-\left(I_{\mathrm{Na}}+I_{K}+I_{A}+I_{L}\right),$$

Here, *C*_m_ = 1 μF/cm^2^ is the capacitance of the membrane, and *V*_m_ is the membrane potential. *I*_shift_ = 8.1 mA is the current used to guarantee the potentials. *I*_Na_, *I*_K_, and *I*_A_ are the ion current components corresponding to the sodium ion (Na^+^), potassium ion (K^+^), and additional K^+^. *I*_L_ is the leakage current component. *I*_Na_, *I*_K_, *I*_L_, and *I*_A_ are related to the membrane potential and corresponding ionic conductance:

(16) $$\begin{array}{l} I_{\mathrm{Na}}=\kappa_{\mathrm{Na}}m^{3}h\left(V_{m}-E_{\mathrm{Na}}\right)\\ I_{K}=\kappa_{K}n^{4}\left(V_{m}-E_{K}\right)\\ I_{L}=\kappa_{L}\left(V_{m}-E_{L}\right)\\ I_{A}=\kappa_{A}A^{3}B\left(V_{m}-E_{A}\right). \end{array}$$

According to Connor’s research [66], the ionic conductance is given by *κ*_Na_ = 120 ms/cm^2^, *κ*_K_ = 20 ms/cm^2^, *κ*_L_ = 0.3 ms/cm^2^, and *κ*_A_ = 47.7 ms/cm^2^ in this paper. Furthermore, *E*_Na_ = 55 mV, *E*_K_ = −72 mV, *E*_L_ = −17.15 mV, and *E*_A_ = −75 mV are the corresponding reversal ionic potentials, as shown in Figure 3. *m*, *h*, *n*, *A*, *B* are the gating variables, and their details can be found in Appendix C. The action potential *V_m_* can be integrated by combining Equations (13)–(16).

### 2.3. Model of Modulation and Perception

**Figure 4:**
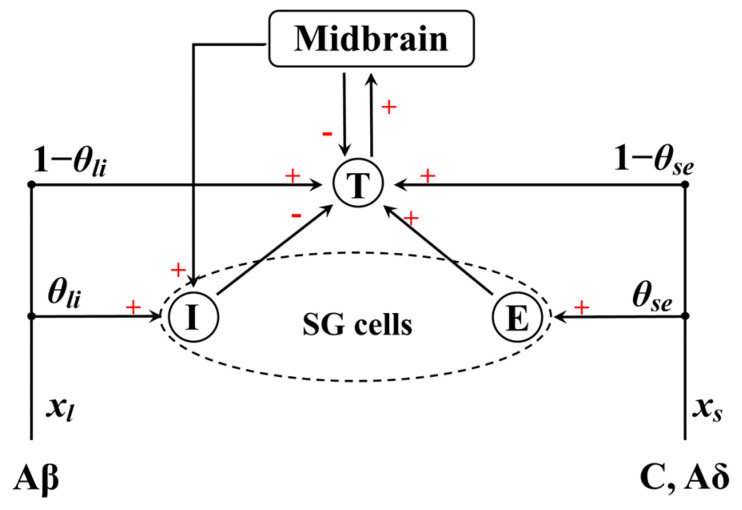
The mathematical model of GCT [67,68,69]. The nerve impulses transmitted through large fibers (Aβ) and small fibers (C and Aδ), with inhibitory and excitatory modulation in SG cells. E, I, and T denote the inhibitory SG cells, excitatory SG cells, and central transmission cells. Symbols “+” and “-” represent excitatory and inhibitory modulation to corresponding cells, respectively.

The mathematical model of gate control theory (GCT) proposed by Britton et al. is introduced to describe the modulation and perception procedure of skin sensations [67,68,69], and is schematically shown in Figure 4.

According to Britton’s theory, the information about noxious stimulus is transmitted through small fibers (C and Aδ), and the information of less intense stimuli is transmitted through the large fiber (Aβ). The stimulus information undergoes inhibitory and excitory modulation when routed through substantia gelatinosa (SG) cells to the central transmission cell (T-cell). This procedure can be expressed by the following equations:

(17) $$\begin{array}{l} \tau \frac{dV_{i}}{dt}=-\left(V_{i}-V_{i0}\right)+g_{li}\left(x_{l}\right)+g_{bi}\left(x_{b}\right),\\ \tau \frac{dV_{e}}{dt}=-\left(V_{e}-V_{e0}\right)+g_{se}\left(x_{s},V_{e}\right),\\ \tau \frac{dV_{t}}{dt}=-\left(V_{t}-V_{t0}\right)+g_{st}\left(x_{s}\right)+g_{lt}\left(x_{l}\right)+g_{et}\left(x_{e}\right)-g_{it}\left(x_{i}\right)-g_{bt}\left(x_{b}\right),\\ \tau \frac{dV_{b}}{dt}=-\left(V_{b}-V_{b0}\right)+g_{tb}\left(x_{t}\right), \end{array}$$

Equation (17) is the general form of the modulation procedure described in GCT, where the subscripts *i*, *e*, *t*, *b*, *s*, and *l* denote the inhibitory SG cells, excitatory SG cells, T-cell, midbrain, small fibers, and large fibers, respectively. The subscript 0 stands for the initial state. *V* and stands for the potential of different cells. *τ* = 0.7, which is a time constant in the equation. *x_l_* and *x_s_* are the impulse frequencies transmitted through large and small fibers, respectively. *x_k_* is linked with *V_k_* by the following:

(18) $$f_{k}\left(V_{k}\right)=-\frac{\left(V_{k}-V_{\mathrm{thr}}\right)}{V_{k0}}\times H\left(V_{k}-V_{\mathrm{thr}}\right),$$

Here, *k* = *i*, *e*, *t*, *b*, *V_k_*_0_ = −70 mV, and *V*_thr_ = −55 mV is the threshold for pain sensation. The function *g_li_* represents the modulation on cell *i* from cell *l*. Equation (17) requires function *g,* a monotonically increasing convergence function. Therefore, the specific expression for Equation (17) is obtained by introducing the hyperbolic tangent function for *g*:

(19) $$\begin{array}{l} 0.7\frac{dV_{i}}{dt}=-\left(V_{i}+70\right)+60\tanh\left(\frac{\theta_{li}x_{l}}{16}\right)+40\tanh\left[f_{b}\left(V_{b}\right)\right]\\ 0.7\frac{dV_{e}}{dt}=-\left(V_{e}+70\right)+40\tanh\left(\frac{\theta_{se}x_{s}}{16}\right)\left\{1+3\tanh\left[4f_{e}\left(V_{e}\right)\right]\right\}\\ 0.7\frac{dV_{t}}{dt}=-\left(V_{t}+70\right)+40\tanh\left[\frac{\left(1-\theta_{se}\right)x_{s}}{16}\right]+40\tanh\left[\frac{\left(1-\theta_{li}\right)x_{l}}{16}\right]\\ \;\;\;+40\tanh\left[f_{e}\left(V_{e}\right)\right]-40\tanh\left[f_{i}\left(V_{i}\right)\right]-40\tanh\left[f_{b}\left(V_{b}\right)\right]\\ 0.7\frac{dV_{b}}{dt}=-\left(V_{b}+70\right)+40\tanh\left[f_{t}\left(V_{t}\right)\right], \end{array}$$

*θ_se_* and *θ_li_* stand for the proportion of the signal that is transmitted through SG cells, which are both taken to be 0.8 in this paper. In this paper, *x_s_* is taken as the frequency of *V*_m_, and *x_l_* is taken to be 0. The signal would be transmitted to the midbrain once the output of T-cell *V_t_* exceeds the *V*_thr_, meaning that *V_t_* is directly related with the level of pain sensation.

## 3. Verification of the Distribution of Stress on Skin

**Figure 5:**
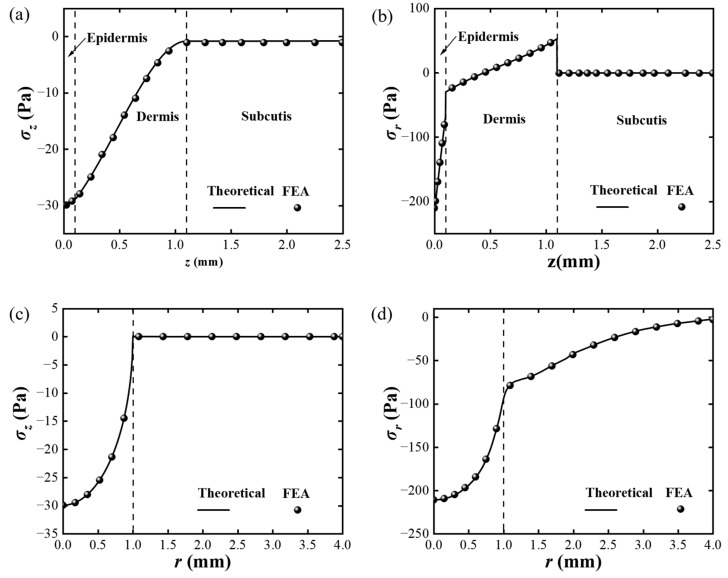
The stress distribution under *p*_1_(*r*) for *σ_z_*, and *σ_r_* along the *z* axis (**a**,**b**) and *r* axis (**c**,**d**). The theoretical and FEA results are shown as lines and dots, respectively. The dash line in (**a**,**b**) represent the boundaries of different layers of skin. And the dash lin in (**c**,**d**) delineate the mechanically-stimulated area. The agreement between the theoretical and FEA data verifies the elastic model of the skin.

Firstly, the mechanical model of skin is verified by comparing the stress distribution with the finite element analysis (FEA). By utilizing the commercialized software ABAQUS 6.14, a 6 mm × 2.5 mm axisymmetric model of skin is established. The Young’s moduli and the thickness of the dermis, epidermis, and subcutis are listed in Table 1 [70]. The bottom surface of the model is fixed. A *p* = 20 Pa, *δ* = 1 mm hemispherical loading *p*_1_(*r*) is applied to the top surface of the model. The model is discretized with 0.005 mm CAX4R mesh, which is enough to guarantee the convergency of the FEA. The variations in two normal stress components, *σ_z_* and *σ_r_*, along the *z*-axis and *r*-axis are denoted by the dots in Figure 5. The theoretical results, shown as the lines in Figure 5, were numerically obtained through the reversed Hankel transform, where the parity of the function is utilized in the integration. In Figure 5a,b, it can be observed that both *σ_z_* and *σ_r_* dramatically decrease when *z* is in the range of the dermis and epidermis, and they converge to 0 at the subcutis. However, *σ_r_* shows a discontinuous change at the interface. Figure 5c demonstrates that the variation in *σ_z_* is consistent with the stress boundary condition, and the Figure 5d shows that *σ_r_* gradually decreases to 0 along the *r*-axis.

**Figure 6:**
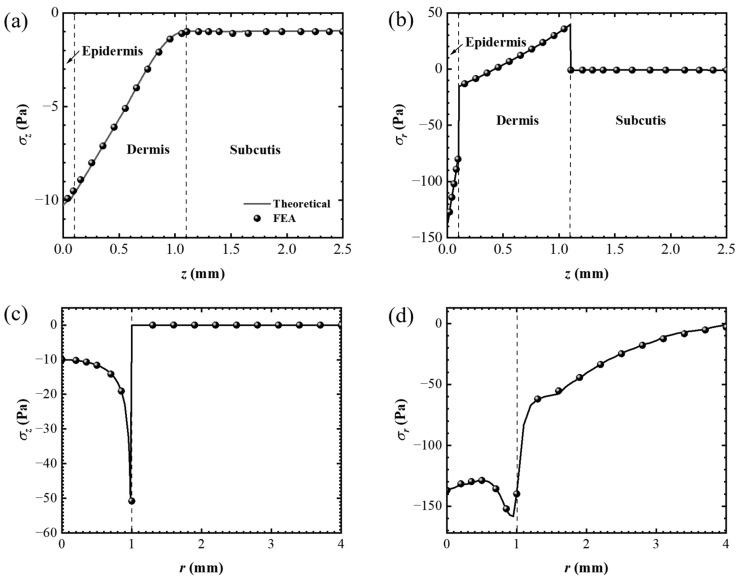
The stress distribution under *p*_2_(*r*) for *σ_z_*, and *σ_r_* along the *z* axis (**a**,**b**) and *r* axis (**c**,**d**).

In addition, the finite element results of the stress distribution under the action of the rigid plate load *p*_2_(*r*) were compared with the theoretical results shown in Figure 6. As shown in Figure 6a,b, the changes in the two normal stress components *σ_z_* and *σ_r_* along the *z*-axis are plotted. Similar to the hemispherical load *p*_1_(*r*), *σ_z_* converges to 0 instantaneously as coordinate *z* increases, and *σ_r_* shows discontinuous changes at the interface of the different layers of the skin. Figure 6c,d show the changes in normal stress components *σ_z_* and *σ_r_* along the *r*-axis. The change in *σ_z_* on the *r*-axis completely corresponds to the stress boundary condition. As it leaves the loading area, *σ_r_* instantaneously converges to 0 along the *r*-axis. The theoretically solved theoretical stress distribution is in good agreement with the FEA results, and the theoretical model of the skin is perfectly verified.

## 4. Case Study

**Figure 7:**
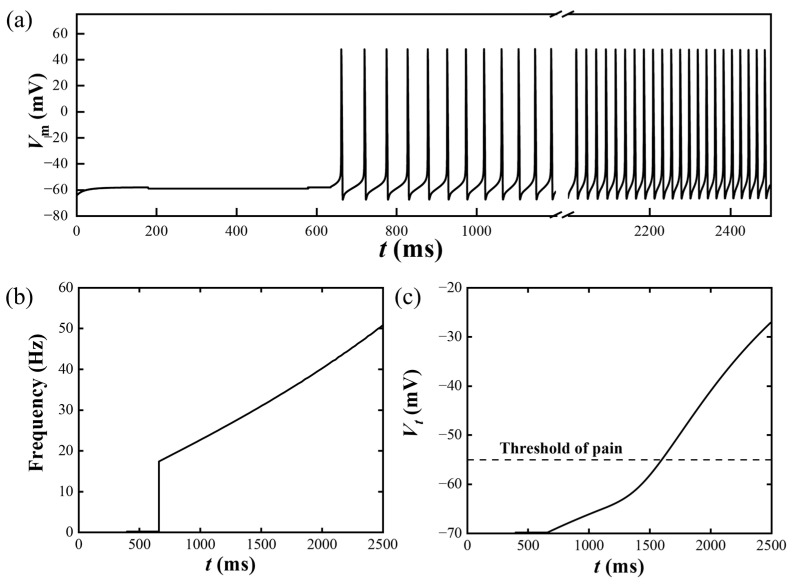
The perception procedure is demonstrated through the application of an increasing hemispherical loading with *p* = 0.4 × 10^6^ *t* (Pa). (**a**) The action potential *V*_m_, (**b**) the frequency of *V*_m_*,* and (**c**) the output of T-cell *V_t_* increase with increasing loading amplitude.

Firstly, the skin sensations experienced under an increasing mechanical stimulus are completely demonstrated in Figure 7. A hemispherical loading with *δ* = 1 mm and an increasing amplitude *p* = 0.4 × 10^6^ *t* (Pa) is applied, where the kinetic effects can be ignored. The nociceptor is assumed to be *z* = 0.5 mm on the *z*-axis. Figure 7a shows the membrane potential induced by the mechanical stimulus; the nerve impulses start after the mechanical stimulus reaches the threshold for pain sensations, *σ*_t_. The frequency of the nerve impulses also increases with the increase in mechanical loading (Figure 7a,b). The output of T-cell *V_t_*, which is directly related to skin sensation, is plotted in Figure 7c. After the formation of nerve impulses gradually increases, uncomfortable pain sensations are perceived after 1500 ms, when *V_t_* exceeds the threshold.

**Figure 8:**
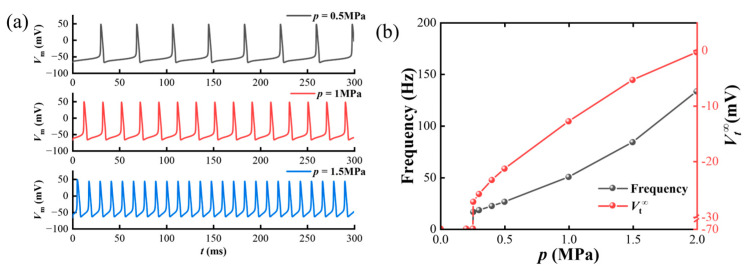
Variations in skin pain sensation under hemispherical loading with different amplitude *p*; (**a**) the variation in membrane potential *V*_m_; (**b**) the variation in *V*_m_ frequency (grey); the *V_t_*^∞^ with *p*, showing that pain perception intensifies with increasing mechanical stimuli.

The effects of different parameters on skin sensation are studied. Firstly, the influence of loading amplitude *p* is analyzed, as demonstrated in Figure 8. A hemispherical loading *p*_1_(*r*) with fixed radius *δ* = 1 mm is applied. In Figure 8a, the variations in *V*_m_ with different loading amplitude *p* (0.5 MPa, 1 MPa, and 1.5 MPa) are plotted. It is clear that the amplitude of *V*_m_ remains stable, but the frequency gradually increases with the loading amplitude. Thus, the relationship between the frequency of *V*_m_, steady state value of *V_t_* (*V_t_*^∞^) and the loading amplitude *p* are plotted as the black line and the red line in Figure 8b. The frequency of *V*_m_ monotonically increases with *p*, which means the intensity of the outside stimuli is carried through the frequency of *V*_m_ [54,71]. Similarly, the *V_t_* is also positively correlated with *p*, suggesting that the pain perception induced by the electronic skin intensifies with increasing mechanical stimuli.

**Figure 9:**
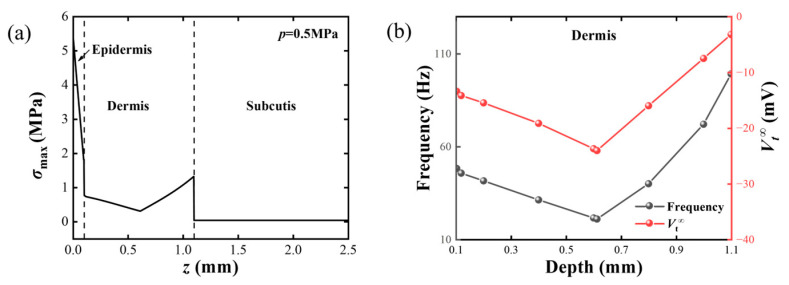
Influence of nociceptor location on skin pain sensation. A hemisphere loading with *p* = 0.5 MPa and *δ* = 1 mm is applied; (**a**) the variation in *σ*_max_ with the depth of the nociceptor in the skin; (**b**) the variation in *V*_m_ frequency (grey) and the *V_t_*^∞^ with *z*.

Secondly, the effect of the nociceptor’s location is analyzed. It is clear that the stress level is different in different locations. A hemisphere loading is applied to the surface of the skin with *p* = 0.5 MPa and *δ* = 1 mm. The variation in *σ*_max_ along the *z*-axis is demonstrated in Figure 9a. The *σ*_max_ shows a dramatic decrease in epidermis, followed by a slight descent and then ascent in the dermis, before finally converging to 0 in the subcutis. According to previous research, the nociceptor is mainly located at the dermis and epidermis [72]. Thus, in Figure 9b, we show the variation in frequency and *V_t_*^∞^ as the nociceptor varies across the dermis. As the nociceptor gradually deepens in the *z* direction, it can be seen that the frequency, which represents the intensity of the external stimuli, first decreases and then increases, which represents the degree of pain sensation, also showing the same trend.

## 5. Phase Diagram of Comfortable Electronic Skin Design

**Figure 10:**
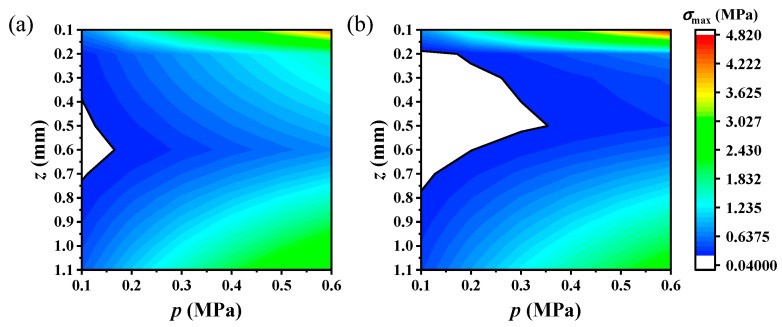
The sensation diagram of human skin under different load sizes and at different nociceptor depths with different mechanical loadings from electronic skins: (**a**) *p*_1_(*r*) and (**b**) *p*_2_(*r*). Different colors correspond to different stress levels *σ*_max_ on the nociceptor. In the blank area, *σ*_max_ is smaller than the stress perception threshold *σ*_t_, meaning that the human body cannot feel the corresponding mechanical stimulation from the electronic skin.

In order to guide and optimize the design of electronic skin and ensure the comfort of potential users, the stress level and the steady state potentials of T cell *V_t_*^∞^ under different loading amplitudes and different nociceptor locations are calculated and summarized. The mechanical loading radius of *p*_1_(*r*) and *p*_2_(*r*) are fixed at δ = 1 mm. Figure 10 shows the variation in stress level *σ*_max_ with the depth of the nociceptor and the load amplitude *p* under (a) *p*_1_(*r*) and (b) *p*_2_(*r*), respectively. Different colors represent different stress levels of *σ*_max_. It can be seen from Figure 10 that *σ*_max_ first decreases and then increases along the *z* direction, and is linearly positively correlated with load amplitude *p*. The maximum value of *σ*_max_ appears at *z* = 0.1 mm, *p* = 0.6 MPa. The interval where *σ*_max_ < *σ*_t_ is marked with a blank area in Figure 10. In the blank interval, the stress level of the nociceptor is less than the stress-sensing threshold, meaning that the mechanically related current component *I*_mech_ would not be induced in the transduction process. And the external mechanical stimuli cannot be sensed by human skin in the blank area in Figure 10.

**Figure 11:**
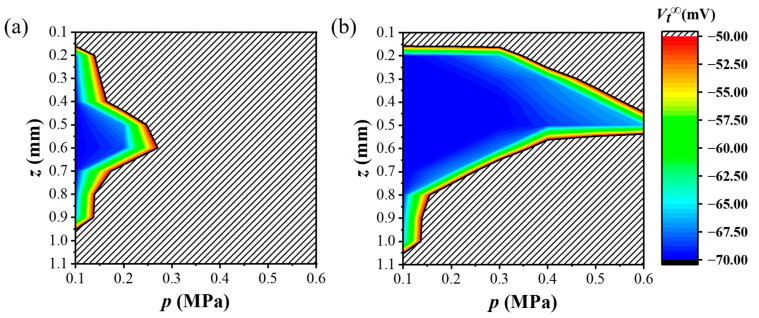
A comfort diagram of human skin under different load sizes and at different nociceptor depths with different mechanical loadings from electronic skins: (**a**) *p*_1_(*r*) and (**b**) *p*_2_(*r*). The potential level steady-state values *V_t_*^∞^ of T cell are denoted by different colors. *V_t_*^∞^ in the shaded area is greater than the pain perception threshold *V*_thr_, exceeding the human pain sensation threshold.

Figure 11 shows how the steady-state potential *V_t_*^∞^ on T cells changes with the nociceptor depth *z* and load amplitude *p* under the mechanical stimuli of *p*_1_(*r*) and *p*_2_(*r*), respectively. Different colors correspond to different *V_t_*^∞^ sizes. The colored intervals surrounded by black solid lines denote the comfort sensation intervals of human skin under mechanical stimulation from an electronic skin, corresponding to a *V_t_*^∞^ less than the pain threshold *V*_thr_ (−55 mV). It can be seen that *V_t_*^∞^ in the color interval in Figure 11 is consistent with the change trend of stress level *σ*_max_ in Figure 10; that is, there is a positive correlation between human comfort perception (*V_t_*^∞^) and *σ*_max_ on nociceptors. In Figure 11, the *V_t_*^∞^ in the gray shaded area is greater than the pain perception threshold *V*_thr_. Mechanical stimulation from electronic skin that falls within the shaded range would lead the human body to experience mechanical pain perception. By combining Figure 10 and Figure 11, the comfort phase diagram that was used to guide the electronic skin design in this article can be obtained. The mechanical comfort of electronic skin could be achieved by controlling the mechanical stimulation amplitude and rationally planning the position where the electronic skin device will be worn.

## 6. Conclusions

This paper presents a novel method for assessing the comfort of electronic skin devices, taking mechanical stimuli into account. The proposed method comprises the following steps: (1) a mechanical model of skin is constructed, where the stress distribution on the skin is obtained theoretically based on elastic theory; (2) the transduction model is developed, where the nervous impulses induced by the mechanical stimuli are acquired through the modified H–H model; (3) the model of modulation and perception is introduced, where the mathematical model of GCT theory is introduced to evaluate the relationship between the nerve impulse and the skin sensation. A mechanical model of skin is verified through a comparison of the theoretical stress distribution and the FEA results. Factors that influence how comfortable an electronic skin are also investigated, including the compression amplitude and the location of the nociceptor. Comfort diagrams are provided to guide the design of electronic skin.

## Figures and Tables

**Table 1:** Mechanical properties and thicknesses for each layer of skin.

	Thickness (mm)	*E* (MPa)	*Ν*
Dermis	0.1	102	0.48
Epidermis	1	10	0.48
Subcutis	1.4	0.01	0.48

## Data Availability

Data are contained within the article.

## Appendix A. Matrix Ψ(*ξ*, *z*)

$$\begin{array}{l} \Psi_{11}=\frac{1}{4\left(1-\nu \right)}[(2-2v-\xi z)e^{-\xi z}+(2-2v+\xi z)e^{\xi z}]\\ \Psi_{12}=-\frac{1}{4\left(1-\nu \right)}[(1-2v-\xi z)e^{-\xi z}-(1-2v+\xi z)e^{\xi z}]\\ \Psi_{13}=-\frac{z}{4\left(1-\nu \right)}[e^{-\xi z}-e^{\xi z}]\\ \Psi_{14}=-\frac{1}{4\left(1-\nu \right)\xi }[(3-4v-\xi z)e^{-\xi z}-(3-4v+\xi z)e^{\xi z}]\\ \Psi_{21}=-\frac{1}{4\left(1-\nu \right)}[(1-2v+\xi z)e^{-\xi z}-(1-2v-\xi z)e^{\xi z}]\\ \Psi_{22}=\frac{1}{4\left(1-\nu \right)}[(2-2v+\xi z)e^{-\xi z}+(2-2v-\xi z)e^{\xi z}]\\ \Psi_{23}=-\frac{1}{4\left(1-\nu \right)\xi }[(3-4v+\xi z)e^{-\xi z}-(3-4v-\xi z)e^{\xi z}]\\ \Psi_{24}=\frac{z}{4\left(1-\nu \right)}[e^{-\xi z}-e^{\xi z}]\\ \Psi_{31}=\frac{\xi^{2}z}{4\left(1-\nu \right)}(e^{-\xi z}-e^{\xi z})\\ \Psi_{32}=-\frac{\xi }{4\left(1-\nu \right)}[(1+\xi z)e^{-\xi z}-(1-\xi z)e^{\xi z}]\\ \Psi_{33}=\frac{1}{4\left(1-\nu \right)}[(2-2v+\xi z)e^{-\xi z}+(2-2v-\xi z)e^{\xi z}]\\ \Psi_{34}=\frac{1}{4\left(1-\nu \right)}[(1-2v-\xi z)e^{-\xi z}-(1-2v+\xi z)e^{\xi z}]\\ \Psi_{41}=-\frac{\xi }{4\left(1-\nu \right)}[(1-\xi z)e^{-\xi z}-(1+\xi z)e^{\xi z}]\\ \Psi_{42}=-\frac{\xi^{2}z}{4\left(1-\nu \right)}(e^{-\xi z}-e^{\xi z})\\ \Psi_{43}=\frac{1}{4\left(1-\nu \right)}[(1-2v+\xi z)e^{-\xi z}-(1-2v-\xi z)e^{\xi z}]\\ \Psi_{44}=\frac{1}{4\left(1-\nu \right)}[(2-2v-\xi z)e^{-\xi z}+(2-2v+\xi z)e^{\xi z}]. \end{array}$$

## Appendix B. Matrix *N*(*ξ*, *z*)

$$\begin{array}{l} N_{11}=\Psi_{11}=\frac{1}{4\left(1-\nu \right)}[(2-2v-\xi z)e^{-\xi z}+(2-2v+\xi z)e^{\xi z}]\\ N_{12}=\Psi_{12}=-\frac{1}{4\left(1-\nu \right)}[(1-2v-\xi z)e^{-\xi z}-(1-2v+\xi z)e^{\xi z}]\\ N_{13}=\Psi_{13}/2G=-\frac{z}{8\left(1-\nu \right)G}[e^{-\xi z}-e^{\xi z}]\\ N_{14}=\Psi_{14}/2G=-\frac{1}{8\left(1-\nu \right)\xi G}[(3-4v-\xi z)e^{-\xi z}-(3-4v+\xi z)e^{\xi z}]\\ N_{21}=\Psi_{21}=-\frac{1}{4\left(1-\nu \right)}[(1-2v+\xi z)e^{-\xi z}-(1-2v-\xi z)e^{\xi z}]\\ N_{22}=\Psi_{22}=\frac{1}{4\left(1-\nu \right)}[(2-2v+\xi z)e^{-\xi z}+(2-2v-\xi z)e^{\xi z}]\\ N_{23}=\Psi_{23}/2G=-\frac{1}{8\left(1-\nu \right)\xi G}[(3-4v+\xi z)e^{-\xi z}-(3-4v-\xi z)e^{\xi z}]\\ N_{24}=\Psi_{24}/2G=\frac{z}{8\left(1-\nu \right)G}[e^{-\xi z}-e^{\xi z}]\\ N_{31}=2G\Psi_{31}=\frac{G\xi^{2}z}{2\left(1-\nu \right)}(e^{-\xi z}-e^{\xi z})\\ N_{32}=2G\Psi_{32}=-\frac{G\xi }{2\left(1-\nu \right)}[(1+\xi z)e^{-\xi z}-(1-\xi z)e^{\xi z}]\\ N_{33}=\Psi_{33}=\frac{1}{4\left(1-\nu \right)}[(2-2v+\xi z)e^{-\xi z}+(2-2v-\xi z)e^{\xi z}]\\ N_{34}=\Psi_{34}=\frac{1}{4\left(1-\nu \right)}[(1-2v-\xi z)e^{-\xi z}-(1-2v+\xi z)e^{\xi z}]\\ N_{41}=2G\Psi_{41}=-\frac{G\xi }{2\left(1-\nu \right)}[(1-\xi z)e^{-\xi z}-(1+\xi z)e^{\xi z}]\\ N_{42}=2G\Psi_{42}=-\frac{G\xi^{2}z}{2\left(1-\nu \right)}(e^{-\xi z}-e^{\xi z})\\ N_{43}=\Psi_{43}=\frac{1}{4\left(1-\nu \right)}[(1-2v+\xi z)e^{-\xi z}-(1-2v-\xi z)e^{\xi z}]\\ N_{44}=\Psi_{44}=\frac{1}{4\left(1-\nu \right)}[(2-2v-\xi z)e^{-\xi z}+(2-2v+\xi z)e^{\xi z}]. \end{array}$$

## Appendix C. Governing Equations for Gating Variables in the Model of Transduction

The gating variable *x* denoting *A*, *B*, x satisfies the following:

$$\tau_{x}\frac{dx}{dt}+x=x_{\infty },$$

*τ_x_* is the voltage-dependent time constant, and *x*_∞_ is the steady-state voltage-dependent function of *x*, which are given by:

$$\begin{array}{l} \tau_{A}=0.3632+\frac{1.158}{1+\exp((V_{m}+55.96)/20.12)},A_{\infty }={\left(0.0761\times \frac{\exp((V_{m}+94.22)/31.84)}{1+\exp((V_{m}+1.17)/28.93)}\right)}^{1/3};\\ \tau_{B}=1.24+\frac{2.678}{1+\exp((V_{m}+50)/16.027)},B_{\infty }={\left(\frac{1}{1+\exp((V_{m}+53.3)/14.54)}\right)}^{4}. \end{array}$$

And for *x* = *m*, *h*, *n*:

$$\tau_{x}=\frac{1}{\alpha_{x}+\beta_{x}},x_{\infty }=\frac{\alpha_{x}}{\alpha_{x}+\beta_{x}};$$

$$\begin{array}{l} \alpha_{m}=\frac{(V_{m}+29.7)/10}{1-\exp(-(V_{m}+29.7)/10)},\beta_{m}=4\exp(-(V_{m}+54.7)/18);\\ \alpha_{h}=0.07\exp(-(V_{m}+48)/20),\beta_{h}=\frac{1}{1+\exp(-(V_{m}+18)/10)};\\ \alpha_{n}=\frac{(V_{m}+45.7)/100}{1-\exp(-(V_{m}+45.7)/10)},\beta_{n}=0.125\exp(-(V_{m}+55.7)/80); \end{array}$$
